# Supplementary material for: Citrus Cell Suspension Culture Establishment, Maintenance, Efficient Transformation and Regeneration to Complete Transgenic Plant
Source: Plants (Basel). 2021 Mar 30;10(4):664. doi: 10.3390/plants10040664 (PMC8066040; doi:10.3390/plants10040664)
Supplement: Supplementary file 1 [file plants-10-00664-s001.pdf]

Table S1. Stock solutions preparation.

No	Chemicals	Amount	Storage	For 1 L media
**BH3 Macronutrient**			4 °C	20 ml
1	KCl	37.5 g/L		
2	$MgSO_4{\cdot}7H_2O$	9.25 g/L		
3	KH_2PO_4	3.75 g/L		
4	K_2HPO_4	0.5 g/L		
**MT Macronutrient**			4 °C	1 L media
1	KNO_3	5.937 g/L		320 ml
2	NH_4 NO_3	5.156 g/L		
3	$MgSO_4{\cdot}7H_2O$	1.156 g/L		
4	KH_2PO_4	0.469 g/L		
5	K_2HPO_4	0.063 g/L		
**MT Micronutrient**			4 °C	1 L media
1	H_3BO_3	1.24 g/L		5 ml
2	$MnSO_4{\cdot}H_2O$	3.36 g/L		
3	$ZnSO_4{\cdot}7H_2O$	1.72 g/L		
4	KI	0.166 g/L		
5	$Na_2MoO_4{\cdot}2H_2O$	0.05 g/L		
6	$CuSO_4{\cdot}5H_2O$	0.005 g/L		
7	$CoCl_2{\cdot}6H_2O$	0.005 g/L		
**MT vitamin stock**			4 °C	1 L media
1	Myoinositol	5 g/L		20 ml
2	Thiamine-HCl	0.5 g/L		
3	Pyridoxine-HCl	0.5 g/L		
4	Nicotinic acid	0.250 g/L		
5	Glycine	0.1 g/L		
**MT calcium stock**			4 °C	1 L media
1	$CaCl_2{\cdot}2H_2O$	29.33 g/L		15 ml
**MT iron stock**			4 °C	1 L media
1	Na_2EDTA	7.45 g/L		5 ml
2	$FeSO_4{\cdot}7H_2O$	5.57 g/L		
**Kinetin (KIN)**			4 °C	1 L media
1	Kinetin (KIN)	0.5 mg/mL		10 ml

Table S2. Media preparation

Media	Preparation steps
Embryonic callus induction media	1. EME– sucrose 0.15 M semisolid medium supplemented with Acetosyringone: 320 mL/L MT macronutrient stock, 5 mL/L MT micronutrient stock, 20 mL/L MT vitamin stock, 15 mL/L MT calcium stock, 5 mL/L MT iron stock, 50 g/L sucrose, 0.5 g/L malt extract, 8 g/L agar, pH 5.8; autoclave medium, add 1 mL/L acetosyringone stock solution to partially cooled medium and pour into 100 × 20 mm petri dishes, 35 mL per dish.<br>2. DOG semisolid medium: Same as EME 0.15 M semisolid medium plus 5 mg/L kinetin (5 mL kinetin stock solution); autoclave medium and pour into 100 × 20 mm petri dishes, 35 mL per dish.<br>3. H+H semisolid medium: 160 mL/L MT macronutrient stock, 20 mL/L BH3 macronutrient stock, 5 mL/L MT micronutrient stock, 20 mL/L MT vitamin stock, 15 mL/L MT calcium stock, 5 mL/L MT iron stock, 50 g/L sucrose, 0.5 g/L malt extract, 1.55 g/L glutamine, 8 g/L agar, pH 5.8; autoclave medium and pour into 100 × 20 mm petri dishes, 35 mL per dish.
Suspension cell culture maintenance media	H+H liquid medium: 160 mL/L MT macronutrient stock, 20 mL/L BH3 macronutrient stock, 5 mL/L MT micronutrient stock, 20 mL/L MT vitamin stock, 15 mL/L MT calcium stock, 5 mL/L MT iron stock, 35 g/L sucrose, 0.5 g/L malt extract, 1.55 g/L glutamine, pH 5.8; pour 500 mL aliquots into 1000 mL glass Erlenmeyer flasks, autoclave and store at room temperature.
Suspension cell regeneration media	1. EME– sucrose 0.15 M liquid medium: 320 mL/L MT macronutrient stock, 5 mL/L MT micronutrient stock, 20 mL/L MT vitamin stock, 15 mL/L MT calcium stock, 5 mL/L MT iron stock, 50 g/L sucrose, 0.5 g/L malt extract. Pour into 250 mL bottles before autoclaving.<br>2. EME– maltose 0.15 M semisolid medium supplemented with antibiotics: 320 mL/L MT macronutrient stock, 5 mL/L MT micronutrient stock, 20 mL/L MT vitamin stock, 15 mL/L MT calcium stock, 5 mL/L MT iron stock, 50 g/L maltose, 0.5 g/L malt extract, 8 g/L agar, pH 5.8; autoclave medium, add 1 mL/L timentin, 1 mL/L cefotaxime and 500 mg/L hygromycin stock solutions to partially cooled medium, and pour into 100 × 20 mm petri dishes, 35 mL per dish.<br>3. EME 1500 semisolid medium supplemented with antibiotics: 320 mL/L MT macronutrient stock, 5 mL/L MT micronutrient stock, 20 mL/L MT vitamin stock, 15 mL/L MT calcium stock, 5 mL/L MT iron stock, 50 g/L sucrose, 1.5 g/L malt extract, 8 g/L agar, pH 5.8; autoclave medium, add 0.5 mL/L timentin, 0.5 mL/L cefotaxime and 500 mg/L hygromycin stock solutions to partially cooled medium, and pour into 100 × 20 mm Petri dishes, 35 mL per dish.<br>4. B+ semisolid medium supplemented with antibiotics: 320 mL/L MT macronutrient stock, 5 mL/L MT micronutrient stock, 20 mL/L MT vitamin stock, 15 mL/L MT calcium stock, 5 mL/L MT iron stock, 25 g/L sucrose, 20 mL/L coconut water, 14.6 mg/L coumarin (10 mL coumarin stock), 0.02 mg/L NAA (200 µl NAA stock), 1 mg/L GA 3 (add 1 mL GA 3 stock solution after medium is autoclaved and cooled to 55 °C in water bath), 8 g/L agar, pH 5.8; autoclave medium, add 0.5 mL/L timentin stock solution to partially cooled medium and pour into 100 × 20 mm petri dishes, 35 mL per dish.<br>5. DBA3 semisolid medium supplemented with antibiotics: 320 mL/L MT macronutrient stock, 5 mL/L MT micronutrient stock, 20 mL/L MT vitamin stock, 15 mL/L MT calcium stock, 5 mL/L MT iron stock, 25 g/L sucrose, 1.5 g/L malt extract, 20 mL/L coconut water, 0.01 mg/L 2,4-D (100 µl 2,4-D stock solution), 3 mg/L BAP (3 mL BAP stock solution); 8 g/L agar, pH 5.8; autoclave medium, add 0.5 mL/L timentin stock solution to partially cooled medium and pour into 100 × 20 mm petri dishes, 35 mL per dish.<br>6. RMAN medium supplemented with antibiotics: 160 mL/L MT macronutrient stock, 2.5 mL/L MT micronutrient stock, 10 mL/L MT vitamin stock, 15 mL/L MT calcium stock, 5 mL/L MT iron stock, 25 g/L sucrose, 0.5 g/L activated charcoal, 8 g/L agar, 0.02 mg/L NAA (200 µl NAA stock solution), pH 5.8; autoclave medium, add 0.5 mL/L timentin stock solution to partially cooled medium and pour into sterile Magenta GA-7 boxes, 80 mL per box.
